# Risks and Benefits of Breastfeeding in COVID-19: Integrative Literature Review

**DOI:** 10.1055/s-0041-1741031

**Published:** 2022-02-09

**Authors:** Ana Clara Alves de Carvalho, Gabriel Campos Carvalhaes Reis, João Guilherme de Moura Oliveira, Raquel Ferreira Borges

**Affiliations:** 1 Departamento de Medicina, Pontifícia Universidade Católica de Minas Gerais, Contagem, MG, Brazil

**Keywords:** COVID-19, SARS-CoV-2 infection, breastfeeding, vertical infectious disease transmission, immunology, COVID-19, infecção por SARS-CoV-2, aleitamento materno, transmissão vertical de doenças infecciosas, imunologia

## Abstract

**Objective** The present article seeks to consolidate existing knowledge on breastfeeding during the SARS-CoV-2 pandemic.

**Data source** Articles from 2020 and 2021 collected from the PubMed, CAPES, Virtual Health Library, Google Scholar, SciELO, and UpToDate databases were analyzed. Books and government documents published in the last decade (2010–2020) were also consulted.

**Study Selection** Sixteen works were used in the present study. The date of publication and discussion of SARS-CoV-2 transmission through breast milk were the inclusion criteria. Thus, articles containing repeated information or with no relevance to add to the production were excluded. Data collection comprised critical reading and synthesis of the main information obtained on the subject, which were performed for the preparation of the present study. The research took place in the period from March 27 to April 2, 2021.

**Synthesis of the data** Breast milk has diverse benefits for both the nursing mother and the infant. The presence of viral RNA by real-time polymerase chain reaction (RT-PCR) in milk from disease-positive mothers has been detected in a few cases, and infant infections in these conditions suggest oral transmission of maternal or third-party origin. The virulence of the novel coronavirus in human milk is not confirmed, while significant amounts of exclusive antibodies are.

**Conclusion** Lactation in the context of COVID-19 has shown greater benefits than risks of vertical transmission. Therefore, it should be encouraged when possible.

## Resumo

**Objetivo** O presente artigo procura consolidar os conhecimentos existentes sobre o aleitamento materno durante a pandemia do SARS-CoV-2.

**Fonte de dados** Foram analisados os artigos de 2020 e 2021 recolhidos dos bancos de dados PubMed, CAPES, Biblioteca Virtual de Saúde, Google Scholar, SciELO e bases de dados UpToDate. Livros e documentos governamentais publicados na última década (2010–2020) também foram consultados.

**Seleção de estudos** Dezesseis obras foram utilizadas no presente estudo. A data de publicação e discussão sobre a transmissão do SARS-CoV-2 através do leite materno foram os critérios de inclusão. Assim, foram excluídos os artigos que continham informações repetidas ou sem relevância para a produção.

**Coleta de dados** Para a preparação do presente estudo, foram realizadas leituras críticas e síntese das principais informações obtidas sobre o tema. A pesquisa ocorreu no período de 27 de março a 2 de abril de 2021.

**Síntese dos dados** O leite materno tem diversos benefícios tanto para a mãe quanto para o lactente. A presença de RNA viral por reação em cadeia de polimerase em tempo real (RT-PCR, na sigla em inglês) no leite de mães positivas foi detectada em alguns casos, e as infecções infantis nestas condições sugerem transmissão oral de origem materna ou de terceiros. A virulência do novo coronavírus no leite humano não está confirmada, enquanto quantidades significativas de anticorpos exclusivos sim.

**Conclusão** A lactação no contexto da COVID-19 mostrou maiores benefícios do que os riscos de transmissão vertical. Por conseguinte, deve ser encorajada sempre que possível.

## Introduction

In the epilogue of December 2019 and in the advent of the year 2020, the world witnessed the rise of severe acute respiratory syndrome (SARS) caused by a new variant of coronavirus – severe acute respiratory syndrome coronavirus 2 (SARS-CoV-2) – which was declared in January 2020 by the World Health Organization (WHO) as an epidemiological pandemic situation. This etiologic agent was responsible for naming a new disease classification known as Coronavirus disease 2019 (COVID-19), which is considered a public health emergency of international concern (EPII). Moreover, this health condition has become one of the main causes of increased mortality rates, as well as of increased efforts by the most diverse centers of medical practice and research in attempts to understand the disease and its therapeutic approach. 1

In general terms, the clinical manifestations of COVID-19 are variable, a reality that demands great attention from healthcare teams. The most common symptoms of the disease are typical of a flu-like syndrome, and involve cough, fever, dyspnea, myalgia, and, in some cases, abdominal pain and diarrhea. So far, it has been observed that the disease most frequently affects adults and the elderly, although it is already known that pregnant women, mothers, newborns, and children can also develop it in its aggravating form. However, it is known that the expressivity of these same aspects can be mild and even asymptomatic, although many cases can evolve to severe acute respiratory syndrome, septic shock, and multiple organ failure. 1 2 In this perspective, the clinical evolution of the disease can be associated to the degree of inflammatory response in the organism of the infected individual, since the higher the degree, the greater the tissue damage and, consequently, the functional compromise of the affected regions. 3

Regarding the transmissibility of the virus, it is known that it enters the body mainly through inhalation of infected air. Moreover, it can enter the body due to contact of the individual with contaminated surfaces (food packaging and other solid objects). 4 In the midst of a scenario of an international health crisis, the debate involving breastfeeding during the pandemic period has arisen in the scientific community. Several publications have already been published in scientific platforms, whose contents investigate not only the possibilities of vertical transmission of the virus through the placenta and breast milk, but also antibody production and its transmission through breastfeeding. Under this bias, the attention in relation to pregnant and postpartum women has increased, since they can also develop COVID-19, especially women in the last trimester of pregnancy. 5

Breastfeeding is described in the literature as a practice that brings benefits to both mother and child. Being exclusive until the first 6 months of life, breastfeeding is able to fully nourish the newborn, promote hydration, transmit antibodies, and develop bonds of affection between the infant and the mother. 6 However, for the purposes of this action to actually be achieved, direct contact between mother and child is essential. In this sense, this reality has brought doubts to the scientific community regarding the transmission of SARS-CoV-2 from the nursing mother to the child, since there is not only a transfer of fluids (breast milk), but also an exchange of aerosols and droplets through the air. Therefore, in a scenario where the etiologic agent of COVID-19 is transmitted mainly by this route, the indication or not of breastfeeding is conflicting. 7

In view of the above, the present work aims to perform a literature review regarding the practice of breastfeeding in the period of the COVID-19 pandemic. This is justified by the possibility of updating and consolidating the knowledge in question.

## Methods

This is an integrative literature review on the relationship between maternal breastfeeding and the transmission of the new coronavirus through this practice. The present study was conducted in Belo Horizonte, state of Minas Gerais, Brazil, in April and March 2021. The guiding question was: “how can SARS-CoV-2 infection affect the practice of breastfeeding, considering its risks and benefits?” Thus, the research was outlined in four stages. In the first, a bibliographic search was conducted in the main databases and through the reading of books, protocols, and guides as complementary material that contemplated the theme for the construction of the study. In the second stage, the articles were selected, which guided the third phase, which was consolidated by the elaboration of a critical review of each material, followed by the writing of the present paper – the fourth stage.

First, the search for articles and scientific research was conducted in the following databases: PubMed, CAPES, Virtual Health Library (VHL), Google Scholar, SciELO, and UpToDate. The keywords *breastmilk* , *breastfeeding* , and COVID- *19* were used in conjunction with the Health Sciences Descriptor (DeCS) *breastfeeding* . Along with these searches, a search was performed in the SciELO database with the keywords COVID- *19* AND *breastfeeding* . In addition, books and other documents from the Ministry of Health were read, all from the last decade (2010–2020).

The selection of studies, in turn, was guided by articles that addressed the evidence on the transmission of SARS-CoV-2 through breast milk and the transmissibility of the virus in question, in addition to prioritizing articles published between the second half of 2020 and the beginning of 2021. In the background, articles were selected from reading their titles and abstracts. Finally, it is worth mentioning that articles containing repeated information or without relevance to add to the production were excluded. A total of 16 works were used.

Therefore, from the selected scientific articles and the complementary material, the third and fourth steps were performed: a critical reading and the synthesis of the main information obtained about the theme for the preparation of the present study. The research took place between March 27 and April 2, 2021, while the discussion of the theme and the preparation of the article took place between April and May, 2021.

## Results

A total of 31 studies were selected from the reading of titles and abstracts. Then, a second choice was performed from the complete reading of the articles, with 12 studies being selected for the production of the work in question. From the total of selected articles, there are 8 review articles; 1 standard operating procedure; 1 comment; 1 prospective study; 1 protocol; 1 epidemiological surveillance guide; 1 food guide, and 1 letter . Besides these, one book on epidemiological approach and materials from the Ministry of Health published in the last decade were complementary to the preparation of the present work. A total of 16 books were used in the preparation of the research and are shown in Table 1 .

**Table 1:** Studies used in the construction of the present article

Title	Authors	Date of publication	Study	Brief description
The Effect of Coronavirus Disease 2019 on Cardiovascular Disease	Askin et al. 1	2020	Review article	The article addresses the pathophysiology of the disease and delves into the risk of its evolution within vascular comorbidities. Finally, it was used for the introduction of the theme and epidemiological contextualization.
Protocolo de Manejo Clínico do Coronavírus (COVID-19) na Atenção Primária à Saúde	Ministério da Saúde 2	2020	Protocol	The protocol addresses the acute condition of COVID-19 within the full clinical spectrum, and provides guidance for therapy.
Severe COVID-19: understanding the role of immunity, endothelium, and	Brandão et al. 3	2020	Review article	The article presents an association between COVID-19 and the endothelial
coagulation in clinical practice				involvement that this health condition causes in this tissue. In addition, it illustrates the immunophysiology of the disease.
Alimentos, Sars CoV-2 e Covid-19: contato possível, transmissão improvável	Franco et al. 4	2020	Review article	The article discusses the possibility of SARS-CoV-2 transmission through food, and was used to contextualize this aspect.
Guia de Vigilância Epidemiológica: Emergência de saúde pública de importância nacional pela doença pelo coronavírus 2019	Ministério da Saúde 5	2021	Epidemiological surveillance guide	The document addresses the epidemiology of COVID-19 by specific age groups, and was used to contextualize the risk scenario for pregnant women from a prevalence and death perspective.
Guia Alimentar para crianças brasileiras menores de 2 anos	Ministério da Saúde 6	2019	Food guide	The document presents a dietary guideline for Brazilian children < 2 years old and objectively addresses breastfeeding, as well as its benefits and an explanation of how to proceed.
[BREASTFEEDING x COVID-19: Elaboration of a SOP for the stages of extraction and storage of breast Milk]	Fernandes et al. 7	2020	Standard operating procedure	The standard operating procedure guides how breastfeeding should be performed in times of COVID-19. Care at the time of breast milk extraction is the main issue addressed.
Guidance on breastfeeding during the Covid-19 pandemic	Calil et al. 8	2020	Review article	The article addresses the importance of breastfeeding, the existing data regarding transmission, the guidelines of the public health agencies, and possible scenarios in the health condition of mother and child to guide the practice under discussion.
Breastfeeding importance and its therapeutic potential against SARS-CoV-2	Vasques et al. 9	2021	Review article	The article presents scientific data regarding the benefits of breastfeeding, especially considering the immunological resources in the context of COVID 19.
Epidemiologia	Gordis L 10	2017	Book	The book was used to support epidemiological concepts in the context of disease transmission. Chapter 2 was used.
Transmission of SARS-CoV-2 through breast milk and breastfeeding: a living systematic review	Centeno-Tablante et al. 11	2021	Review article	The paper is a literature review of evidence of SARS-CoV-2 in breast milk. The authors address several studies that present statistical data and health conditions of mothers and children on COVID-19 and breastfeeding.
Susceptibility to COVID-19 in pregnancy, labor, and postpartum period: immune system, vertical transmission, and breastfeeding	Vale et al. 12	2021	Review article	The article addresses questions about the pathophysiology of COVID-19, especially in pregnant and postpartum women. They discuss a little about vertical transmission, and put the focus of research on the guidelines for mothers and children in the postpartum period.
Covid-19 and breastfeeding: what's the risk?	Hand et al. 13	2020	Comment	The paper affirms the numerous benefits that breastfeeding promotes in the health of mother and child. In addition, statistical data on the presence and transmission of SARS-CoV-2 in breast milk is also emphasized.
Breastfeeding during the COVID 19 pandemic – a literature review for clinical practice	Lubbe et al. 14	2020	Review article	The study, with a clinical practice oriented approach, addresses the possible scenarios regarding mother and child conditions in the context of COVID-19. It also highlights data on the transmission of SARS-CoV-2 through breast milk.
Characterization of SARS-CoV-2 RNA, antibodies, and neutralizing capacity in milk produced by women with COVID-19	Pace et al. 15	2021	Prospective study	The article presents a study conducted in mothers who were infected with SARS-CoV-2, discussing the presence of virus RNA, specific antibodies, and neutralizing ability against the novel coronavirus in the breast milk samples tested.
Antibodies in the breast milk of a maternal woman with COVID-19	Dong et al. 16	2020	Letter	The paper presents a case report that takes into consideration the clinical findings in puerperae and neonates who tested positive for COVID-19. The focus of the discussion was on breastfeeding.

## Discussion

### The Benefits of Breastfeeding

There is a consensus in the literature that breastfeeding designates benefits for both the nursing mother and the infant. These advantages can be attested to by increased quality of life and reduced maternal and infant morbidity and mortality.

In this context, there is evidence that reinforces the significant decrease in the incidence of diseases, linked, above all, to poor prognoses in the neonatal, postnatal, and childhood phases. In line with this fact, it is observed that lactation is responsible for increasing the intellectual coefficient and preventing dental bone disorders, allergies, and chronic conditions in adolescence and adulthood. 8 9

Another point widely discussed in all the studies analyzed is the immunological, anti-inflammatory, and anti-infective properties of natural food from the mother. Thus,

breastfeeding in the context of COVID-19 is an important resource to control the increase in virulence, 9 which is the ability of the etiologic agent to cause infection. 10 Regarding immunological benefits, breast milk has antimicrobial properties. Moreover, it is primarily responsible for the 48% decrease in necrotizing enterocolitis in infants, highlighted in a cohort study. 9

The antimicrobial, anti-inflammatory, and immune system modulating actions can be conferred by mucin, lactalbumin, lactadherin, lactoferrin, amino acids, casein, and tryptophan, present in breast milk. These substances promote activation and proliferation of phagocytic cells, such as macrophages, dendritic cells, and apoptotic cells. In addition, they mediate the development of cells that secrete cytokines and regulatory proteins of the cell cycle and cell division that are important in the response of the inflammatory process. 9

The proliferation and activation of B and T lymphocytes are also ensured by breast milk through the release of immunomodulatory peptides. Casein, lactalbumin, and tryptophan are notable nutrients in this role. The latter is also related to the prevention of chronic conditions involving the gastrointestinal tract. Furthermore, these components act on the child's development by modulating the secretion of metabolic precursors. 9

The protection of the intestinal mucosa by barrier formation, the proliferation and maturation of tissue cells, and the homeostasis of the microbiota are modulated by the components of breast milk. In this sense, mucin, casein, lactoferrin, amino acids, and tryptophan are mainly responsible for this effector mechanism. Therefore, it is observed that these nutrients create a favorable environment for the growth and development of the beneficial intestinal microbiota, which prevents infections and inflammation by reducing inflammatory cytokines. In addition, they regulate the motility of the gastrointestinal tract. 9

Lactadherin and lactoferrin also play an important role in antiparasitic actions and in the neutralization of antigens, preventing their colonization. These substances promote the secretion of interleukin-10 and transforming growth factor beta (TGF-β) by regulatory T lymphocytes, which are cell adhesion molecules that inhibit binding to pathogens. The latter, in particular, has shown promising results regarding the neutralization of cell receptors, which may confer prevention of COVID-19. 9 10

The presence and transfer of specific antibodies, including those compatible with SARS-CoV-2, are also important points to note in favoring immune competence. Immunoglobulins A (IgA) have been identified in the breast milk of infected mothers, which can be transferred to their children and remain reactive for a period of ∼7 months. These are important antibodies for preventing severe cases of the disease, as well as respiratory and gastrointestinal disorders. Thus, the immunological benefits outweigh the risks of breastfeeding due to the presence of these peptides, considering also a possible pre-exposure to the virus by mother-child contact. 8 9 11 12

Added to this, the increased expression of type I interferon in infants is related to the practice of breastfeeding. This importance is consolidated in the benefits for fighting COVID-19, analyzed in two studies in which poor prognosis of this new disease in patients in whom the deficiency of this protein is conditional. 13

Notwithstanding the benefits to the infant, breastfeeding contributes to the health of the mother. According to some studies, 8 it is possible to observe that such an act causes a decrease in postpartum bleeding, aids in weight loss, and is a natural contraceptive method. Moreover, they highlight its preventive potential against breast and ovarian cancers, type II diabetes mellitus, and postpartum depression.

It is also important to highlight that maternal breastfeeding is related to the bond between mother and child. This condition causes an increase in the quality of life by promoting the mental health of the puerperal woman, especially in the period of social

distance which is conferred. 9 Therefore, the separation of the two parties can be associated to adverse effects on mental health. 14

The risks associated with early weaning, which is characterized by the interruption of lactation before the first 6 months of life, are discussed. Thus, lower incidence of infections are observed; hospitalization rates decreased by 5% per month of breastfeeding; and increased proliferation and differentiation rates of cells and biomolecules responsible for antigenic protection barriers were observed. Furthermore, it was shown that breastfeeding time is indirectly related to the proportion of morbidity and mortality and serious conditions. Thus, it is concluded that the absence of exclusive breastfeeding can serve as a “gateway” to infections, especially those caused by SARS-CoV-2, in addition to causing intestinal inflammation. 9

To reaffirm the benefits, the analyzed studies assess that the use of formula as a substitute should be avoided. In this context, it is important to point out that errors associated with the handling of formulae cause absence in the necessary nutritional coverage. The presence of coagulant proteins that hinder digestion and cause a decrease in immunological power when compared to natural food should also be highlighted. From this perspective, the use of formula is also associated with the development of milk protein allergies. 8 13

Finally, issues related to economics and availability should be considered in the discussion. Regarding breast milk, it is a clean, safe, complete, ready, and easily accessible food. Moreover, the high cost involved in the purchase of formula, as well as the possibility of its shortage in the current context, is not a reason for concern on the part of nursing mothers and their families. In this context, an analysis of the absence or interruption of breastfeeding designated the loss of 341.3 billion USD annually, when considering the maternal and infant morbidity and mortality that could be avoided, the cost of purchasing the substitute for human milk, and cognitive loss. 8 9 10 11 12 13

In view of the above, it is concluded that breastfeeding brings significant benefits to the mother and the child. Thus, on May 27, 2020, the World Health Organization (WHO) updated the Interim Guidance on Clinical Management of COVID-19. In this decision, the institution reaffirmed that the benefits of this exclusive practice up to the first 6 months and complementary up to 2 years old outweigh the risks of maternal-to-child transmission of SARS-CoV-2 through human milk and poor outcomes of COVID-19 in infants and newborns. 11

### Evidence for Sars-Cov-2 in Milk, Vertical Transmission, Antibodies, and Viral Neutralization

In 37 studies, 19 out of 77 evaluated children were diagnosed with COVID-19 by viral RNA and antibody detection. 11 Of these, 10 were certainly fed with breast milk. In another study with 79 samples, several infants tested positive for RT-PCR SARS-CoV-2 were exclusively fed milk that was negative for the same test. 11 All had contact with COVID-19-positive third parties. Furthermore, out of the 77 mothers analyzed, 59 of them tested negative for RT-PCR. Another relevant point is that three positive milk samples were collected during the symptomatic phase of the mother. Another 43 negatives were collected during the symptomatic or convalescent phase. This indicates the possibility that SARS-CoV-2 is not transmitted through breast milk during the acute phase, when maternal viremia is expected to be higher, or during the convalescent phase. After 4 to 5 weeks of infection, no milk samples positive for SARS-CoV-2 were identified, indicating that the alveoli do not denote a viral reservoir. 11

Importantly, the aforementioned research has limitations, since the milk extraction and analysis process were not detailed, which may suggest exogenous contamination. In addition, it is not possible to estimate the virulence of breast milk, since the viral culture technique was not tested; similarly, it is worth pointing out another study that showed the impossibility of culture from detected SARS-CoV-2. It is also important to investigate the possibility of this condition changing during lactation, since breast milk changes at different stages. 11

From maternal collections, the main hypothesis of infection and transmission between mother and child is orally or through infected instruments, suggesting that lactation is safe when it comes to virulence. The mother should be aware of the existing data regarding vertical and breastfeeding transmissions, besides the fact that there are newborns who become infected, probably by the oral route, but have a good prognosis. Thus, personal protective measures and breast hygiene should be strictly followed. Moreover, the researchers do not state that there is a possibility of vertical transmission, but they also do not rule out this possibility. 12

Influenza was used to exemplify how breastfeeding can contribute to protect the child if the mother is infected. In this context, the passage of IgA is possible through breastfeeding and can remain in the organism of the infant for up to 6 months. Another important point to be discussed is that breastfeeding provides increased type I interferon in infected individuals, which classifies such a response as innate antiviral to influenza, and not to respiratory syncytial virus or to human metapneumovirus. 13

A study of 6 women after their first lactation, for example, revealed 100% negative results for detection of SARS-CoV-2 viral RNA by RT-PCR. In addition, another survey of 19 women also failed to demonstrate the presence of the virus in maternal samples. In contrast, SARS-CoV-2 secretory immunoglobulin A (SIgA) was found to be present in the milk of 80% of mothers previously infected with the SARS-CoV-2. These analyses have also shown that, despite obtaining negative results in PCR test (polymerase chain reaction) specific for SARS-CoV-2, there is presence of these immunoglobulins in breast milk. 13

It was also possible to identify SARS PCR-positive maternal samples from three different women. However, the results obtained were negative on days 3, 14 and 14, respectively. Like the mothers, the children in the study tested positive for the viral gene, despite their good clinical condition. Furthermore, it cannot be said that the infection was caused by the virus present in the milk. The authors of this study state that it is still difficult to confirm the possibility of mother-to-child perinatal transmission through breastfeeding, and that it may occur rarely. 13

Not far from this scenario, it is necessary to point out that, in Italy, two newborns had positive pharyngeal swab results for SARS-CoV-2 days after birth, but one of them was asymptomatic and the other had mild symptoms. From the analysis of the breast milk with negative result for SARS-CoV-2, the hypothesis was that a third person infected the mother and the newborn at the same time after birth. Another episode is that of a 13-week-old Chinese newborn who – after presenting with weak signs of COVID-19–tested positive for the presence of viral RNA, as did his mother. Breast milk samples did not detect the presence of the virus, but rather of antibodies against COVID-19. It is noteworthy that both the mother and the newborn were exposed to a third person, who also tested positive for the disease, suggesting that the infection occurred through the oral route. 13

Following up on the discussion about immunoglobulins, a prospective study – with a repeated measures longitudinal design – analyzed 18 women who had COVID-19 confirmed by laboratory diagnoses. With this, 37 human milk samples were collected and analyzed, and none of them had detectable SARS-CoV-2 RNA. In addition, 76% of the breast milk samples had IgA for SARS-CoV-2, and 80% of these samples contained class G immunoglobulins (IgG) for the same virus, with concentrations of the former being higher compared with the latter. Another important point is that 62% of the samples were able to neutralize the virulence of the etiologic agent in vitro, in contrast with samples collected prior to the pandemic, none of which showed such potential. Finally, 70 breast swabs were tested and only 8 showed evidence of SARS-CoV-2 RNA. 15

The results of the analyzed studies suggest that breastfeeding does not transmit SARS-CoV-2 from the mother with mild to moderate COVID-19 symptoms to her child. However, transmission through the skin of the breast is possible. It is noteworthy that no SARS-CoV-2 RNA was found in the breast after washing, evidencing that individual protection measures during breastfeeding and milk extraction reduce the rate of contamination to the infant. Furthermore, the literature shows that the concentrations of immunoglobulins specific for SARS-CoV-2 are directly linked to the viral neutralization potential. Therefore, these results may serve as a rationale encouraging the continuation or initiation of breastfeeding in women with nonsevere COVID-19. 15

To exemplify the data in question, it is suggested to approach a study built from the follow-up of a mother who tested positive for COVID-19 and her son, who has high rates of anti-SARS-CoV-2 class M immunoglobulin (IgM), which highlights the possibility of antibody transfer through breastfeeding. It was pointed out that the mother, still pregnant when she was admitted to the hospital, was symptomatic for COVID-19 and, therefore, underwent imaging and molecular testing in an attempt to make a diagnosis. X-rays were taken, which revealed irregular ground-glass opacities in the left lung, consistent with clinical management protocols for this acute condition. The positive smear for RT-PCR was obtained from the upper airway. 16

The delivery of the approached pregnant woman occurred under normal conditions, with all properly paramented. The newborn presented with excellent birth conditions, and soon after delivery, a pharyngeal sample was collected for molecular testing, which tested negative. High levels of IgA anti-SARS-CoV-2 were found in the maternal milk, which denotes the protective nature of the milk. Immunoglobulin G was also detected in the newborn, but with a decline after one and a half months. 16

In a similar vein, another study establishes the modulation of the imune in COVID-19. In this study, 100% of mothers who had recovered from COVID-19 had developed SIgA specific for SARS-CoV-2, and 99% of these proteins were reactive for the peak protein of the virus in another smaller assay. These immunoglobulins can remain in breast milk 7 months after birth and confer prevention of COVID as well as decreased symptoms in the 1 ^st^ year of life if the infant becomes infected. In addition, they are optimal for recovering severe cases of the disease, are resistant to proteolysis, and may confer protection from respiratory and intestinal diseases. Thus, breastfeeding may protect against SARS-CoV-2 infection at a level that lessens the impact on the intestinal mucosa and its severity. Accordingly, research has shown that cessation of this exclusive practice can serve as a “gateway” to infections, particularly those caused by SARS-CoV-2, in addition to causing intestinal inflammation. 9

Therefore, we conclude that there is no relevant evidence of the presence of SARS-CoV-2 in breast milk by means of the RT-PCR laboratory test and that, in the positive samples, it was not possible to perform viral culture to evaluate the infectious capacity, so that virulence is not observed. This fact suggests that the main means of transmission of SARS-CoV-2 from mother to infant is still oral, and that human milk is a means of passive immunization, since it presents antibodies against the pathogen. Therefore, it is evident that the benefits of breastfeeding outweigh the possible risks of contamination by SARS-CoV-2 and that this practice should be encouraged, but with a shared decision between mother, family members, and health professionals.

## Conclusion

In the present study, the relationship between breastfeeding and the potential for transmission of SARS-CoV-2 during the current pandemic scenario was evaluated. Thus, it was observed that the benefits provided by lactation, both for the mother and the infant, outweigh the risks associated with this practice in COVID-19-positive women. In this context, little evidence of SARS-CoV-2 in human milk from COVID-19- positive mothers has been observed. Furthermore, infection in children and in neonates suggests oral transmission, since the virulence of natural food has not been confirmed. It is important to note that antibodies unique to SARS-CoV-2 were detected in the analyzed samples. Finally, it can be concluded that, based on evidence suggesting the impossibility of transmission of SARS-CoV-2 through breast milk, breastfeeding should be encouraged. In this process, however, the autonomy, the desire, and the well-being of the mother and of the child must be assured during the decision-making process, which must be indispensably shared.
